# Juvenile Xanthogranuloma: A Visual Clinical Diagnosis

**DOI:** 10.7759/cureus.98625

**Published:** 2025-12-07

**Authors:** Raquel Santos, Ana Marta Barros, Marisa Carvalho

**Affiliations:** 1 Pediatrics, Unidade Local de Saúde de Trás-os-Montes e Alto Douro, Vila Real, PRT; 2 Pediatrics, Unidade Local de Saúde de Gaia e Espinho, Vila Nova de Gaia, PRT; 3 Pediatrics and Neonatology, Unidade Local de Saúde de Trás-os-Montes e Alto Douro, Vila Real, PRT

**Keywords:** case report, diagnosis, juvenile xanthogranuloma, non-langerhans histiocytosis, pediatric dermatology

## Abstract

Juvenile xanthogranuloma (JXG) is a benign non-Langerhans cell histiocytosis that typically presents during infancy with solitary or multiple yellow-orange papules or nodules. We describe a 10-month-old male infant who presented with multiple progressively appearing yellow-orange papular lesions since six months of age. Dermatology and ophthalmology evaluations showed no evidence of ocular or systemic involvement. The clinical presentation was characteristic of JXG, and due to its benign nature and parental preference, no biopsy was performed. At 13 months of age, the lesions showed clear spontaneous regression. This case highlights the importance of recognizing the classic clinical features of JXG, which allows for a confident clinical diagnosis and the avoidance of unnecessary invasive procedures.

## Introduction

Juvenile xanthogranuloma (JXG) is the most frequent type of non-Langerhans cell histiocytosis in children, usually presenting during infancy or early childhood as solitary or multiple yellow-orange papules or nodules [1]. Its diagnosis is primarily clinical in typical cases, and most lesions undergo spontaneous regression without requiring treatment [2]. Despite its benign course, ocular involvement may occur in a minority of patients, such as nodular or diffuse iris lesions (secondary complications such as hyphema and glaucoma), and yellowish masses on the conjunctiva, corneoscleral limbus, or eyelid. Therefore, an appropriate ophthalmological evaluation is essential. This report describes a case of multiple cutaneous JXG lesions diagnosed clinically, emphasizing the characteristic features that support recognition and reassurance without unnecessary diagnostic interventions.

## Case presentation

**Figure 1:**
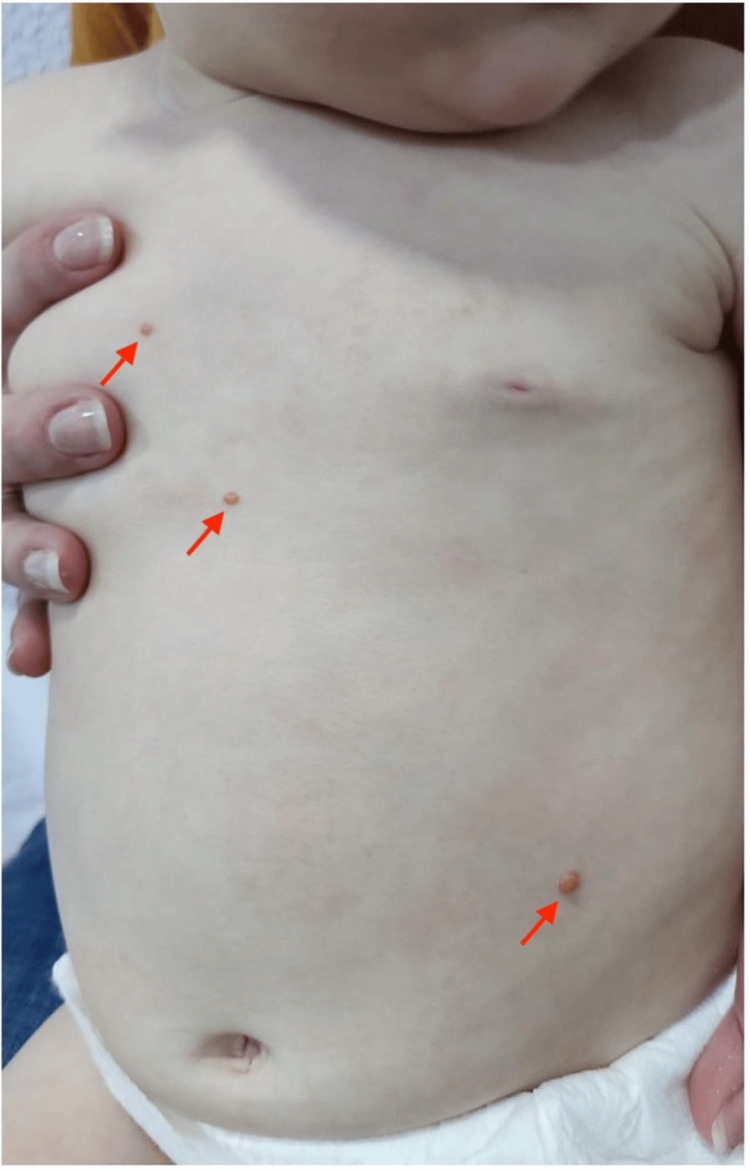
Multiple yellow-orange papules on the anterior trunk (arrows), clinically consistent with juvenile xanthogranuloma.

**Figure 2:**
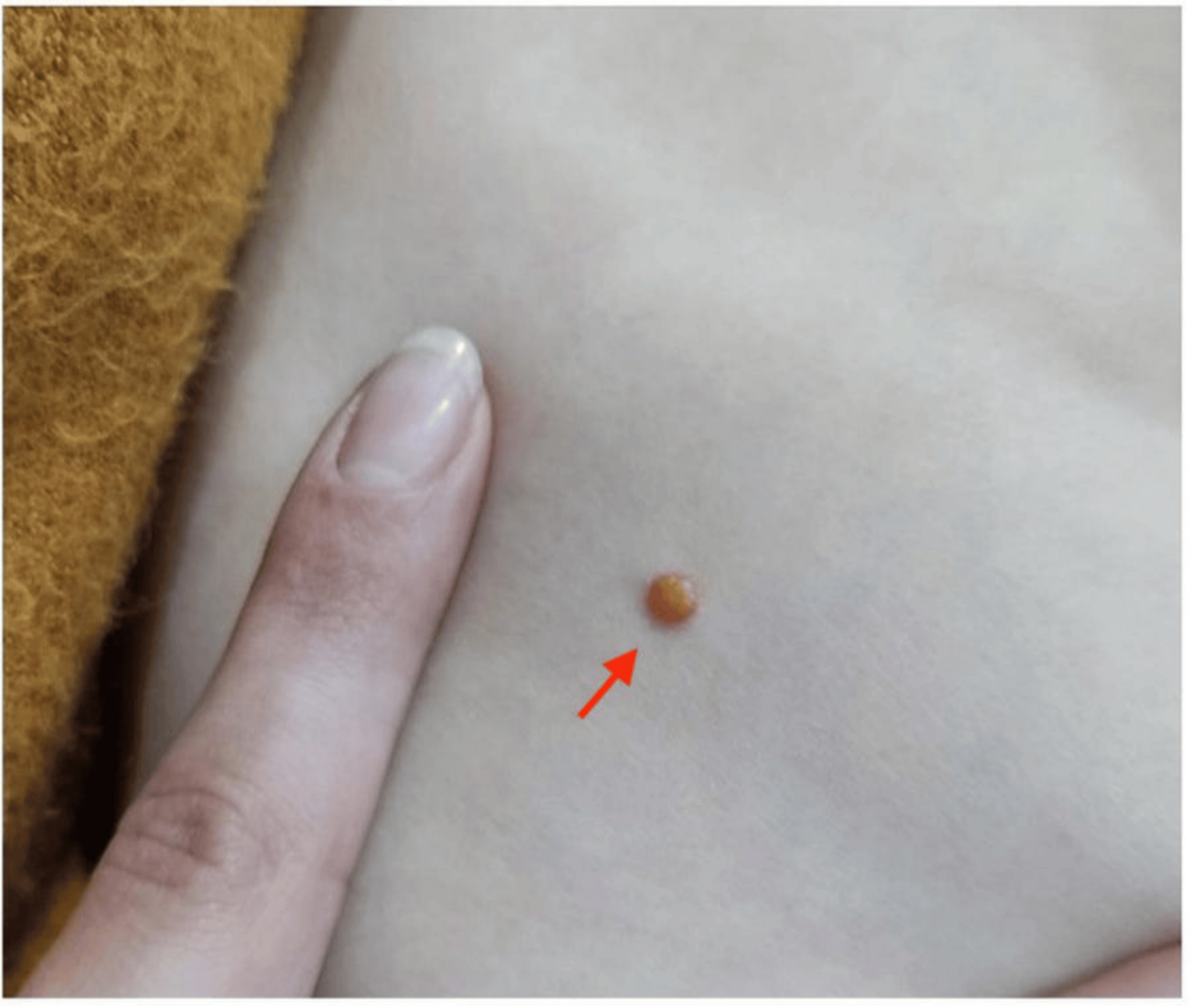
Close-up of a dome-shaped papule with an orange hue and smooth surface, characteristic of juvenile xanthogranuloma.

A 10-month-old male infant was evaluated in the emergency department for multiple papular skin lesions. The lesions had first appeared at six months of age, gradually increasing in number. Upon examination, six well-defined papules with a yellow-orange hue were observed: three on the anterior trunk, one millimetric lesion on the right temporal region, one on the upper back, and one on the eyelid (Figures 1-2). No associated erythema, ulceration, or tenderness was noted. The lesions were firm but mobile upon palpation.

The pediatric dermatology consultation considered the lesions highly consistent with JXG based on morphology and distribution. A pediatric ophthalmology evaluation was performed to exclude ocular involvement, and no abnormalities were identified.

**Figure 3:**
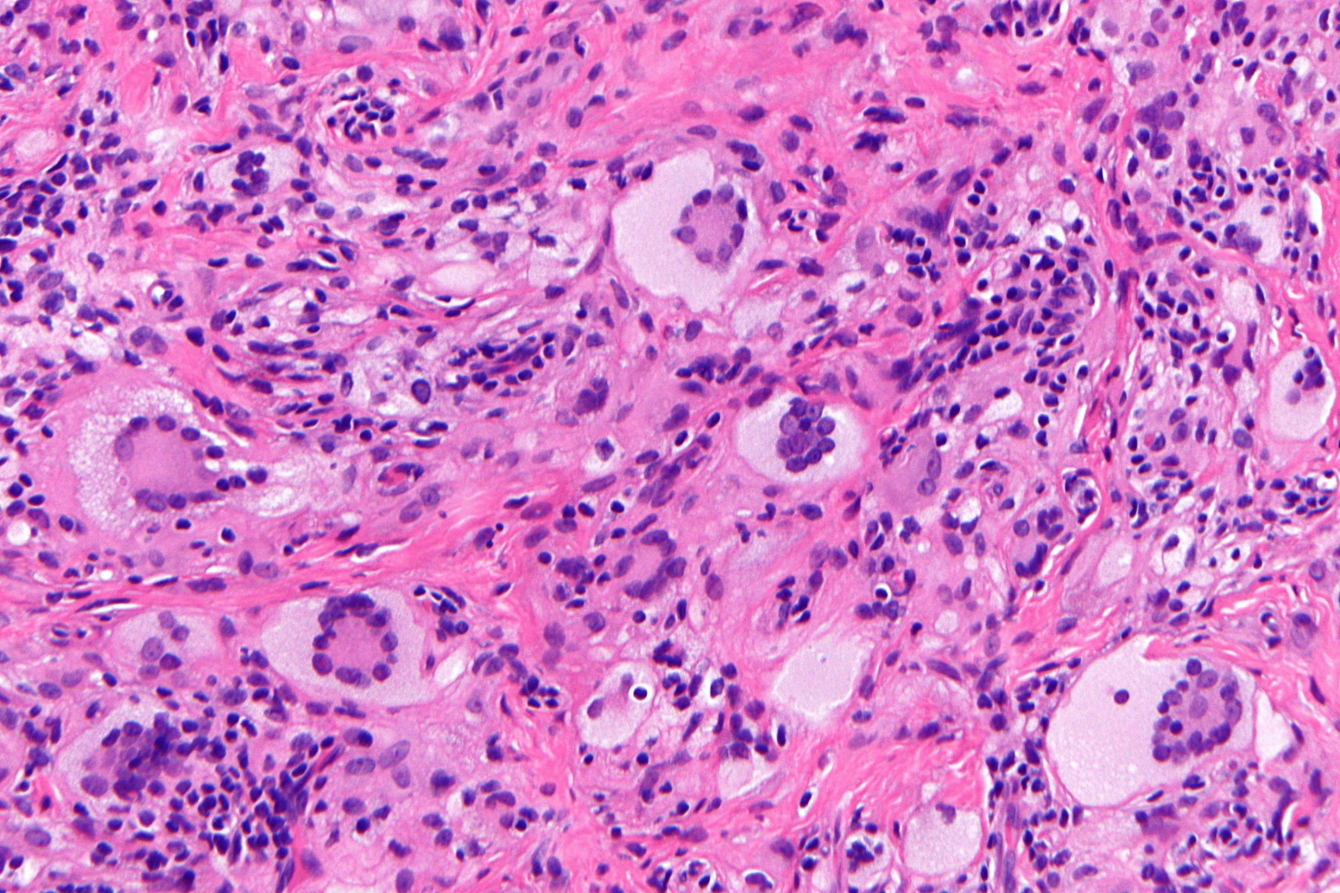
Representative histopathological features of juvenile xanthogranuloma (H&E stain) demonstrating foamy histiocytes and characteristic Touton-type giant cells. Credit: Image reproduced from Wikimedia Commons (author: KGH) under the Creative Commons Attribution-ShareAlike 3.0 License; included for reference as no case-specific biopsy was performed.

Given the typical clinical presentation, benign natural history, and parental preference, a biopsy was not performed. As histopathological imaging was not available, a representative open-access histopathological image illustrating typical features of JXG has been included for reference (Figure 3).

The family was informed about the expected course of the disease, including its tendency for spontaneous resolution. At 13 months old, the lesions showed clear signs of regression (progressively reduced). No systemic involvement was detected. The patient remains under clinical follow-up.

## Discussion

JXG represents the most common form of non-Langerhans cell histiocytosis in childhood and typically affects infants and young children [1]. Although solitary lesions are more common, multiple lesions occur in a significant minority of cases and may prompt evaluation for systemic involvement [2]. Classic clinical features include well-defined, firm, dome-shaped papules or nodules with a characteristic yellow-orange coloration due to lipid-laden histiocytes.

Diagnosis is usually clinical when the presentation is typical, as in this case. Dermoscopy may aid diagnosis, but histopathologic confirmation is not mandatory unless lesions are atypical, rapidly progressive, or associated with systemic findings. Avoiding unnecessary biopsies is particularly relevant in infants, given the benign and self-limited nature of the condition.

Ocular involvement, though rare, is the most significant potential complication of JXG and warrants screening in patients with multiple lesions or early-onset disease [3]. Early ophthalmologic evaluation is therefore recommended. Systemic involvement is uncommon but has been described, particularly in patients with numerous cutaneous lesions.

The natural history of JXG is favorable, with spontaneous involution of skin lesions occurring over months to years. Recurrence is rare, and long-term prognosis is excellent. This case adds to existing literature demonstrating that careful clinical assessment and appropriate referral allow for safe, noninvasive management.

## Conclusions

JXG can often be diagnosed clinically based on its characteristic cutaneous features, especially in infants. Recognition of these features is essential to avoid unnecessary invasive diagnostic procedures. Although rare, ocular involvement must be excluded through appropriate referral. This case illustrates a typical presentation of multiple cutaneous JXG lesions with spontaneous regression, reinforcing the benign nature and excellent prognosis of the condition.
